# Left behind places, neoliberalism and systemic violence in the UK

**DOI:** 10.3389/fsoc.2025.1632190

**Published:** 2025-07-23

**Authors:** Luke Telford

**Affiliations:** School for Business and Society, University of York, York, United Kingdom

**Keywords:** left behind places, neoliberalism, capitalism, systemic violence, inequality

## Abstract

Characterized by structural problems including persistent deprivation, the UK's left behind places have attracted increased scholarly, political and media attention in recent years. Throughout neoliberalism governments have implemented a range of policies to attempt to address the plight of these locales, but successful attempts at turning around their socio-economic predicaments are rather rare. One fundamental problem is that the UK has been a low investment nation across much of the neoliberal era, resulting in left behind zones not receiving the level of resourcing required to ameliorate the issues they face. This article begins by outlining how the decline of left behind places is tethered to neoliberal political economy, before discussing neoliberalism's failure to resurrect these areas. The paper then theoretically explicates left behind places in relation to systemic violence and absence. It suggests neoliberalism's inability to revive the left behind is systemically violent in its effects, resulting in a sense of political invisibility and the loss of hope. The article closes by claiming the absence of widespread political representation, engagement and a positive future among the left behind ensures the continued infliction of systemic violence.

## Introduction

UK capitalism's social democratic phase (1945–79) represented a period of relative stability and security, with the citizenry enduring unprecedented gains in their living standards (Streeck, 2016). Consecutive governments generally governed with the class compromise in mind (Wistow, 2022; Wolf, 2023), involving the nationalization of key industries, palpable trade union power, a national health service free at the point of use and a universal welfare state that hinged upon fighting against Beveridge's (1942) five giants of idleness, ignorance, disease, squalor and want. These policies were combined with a redistributive regional policy that sought to rebalance the economy away from overheated London, involving controls on new investment in parts of the southeast and government grants and loans for businesses who set up in development areas especially in the North (Martin, 2024). As such, inequalities between places were kept at socially acceptable levels (Dorling, 2024). Regardless of where an individual lived, there were also feelings of relative optimism and hope of a better future (Hall and Winlow, 2025; Streeck, 2016).

UK capitalism, however, encountered structural crises in the 1970s. This particularly included stagflation—high inflation accompanied with stagnant economic growth and rising unemployment—as well as the 1978–79 winter of discontent. Such socio-economic predicaments represented what Martin et al. (2021, p. 30) described as a “hinge of history”. The post-war settlement collapsed under the weight of its own contradictions, replaced by neoliberal capitalism. Neoliberalism particularly entailed a transformative shift from an industrialized to post-industrial economy (Hudson, 2022; Winlow, 2025), involving an ever-widening economic gap between the rich and poor, especially across affluent and deprived places (Dorling, 2024). Over the past half a century or so, geographical inequality has intensified by around 50% (Martin et al., 2021), with some locales evolving into *affluent areas of contentment* and others becoming *left behind*.

It is widely known that the UK's regional inequalities are now arguably the worst in the advanced economies (Fai and Tomlinson, 2023; McCann, 2016). Where you live has a palpable bearing on your opportunities in life, outcomes and prospects for upward social mobility (Dorling, 2024). Whilst most places containing *promising prospects* are found in London and the surrounding Home Counties, the least favorable are particularly located in Northern areas regarded as left behind including Hull, Middlesbrough and Stoke-on-Trent (Social Mobility Commission, 2024). In recent years, these types of areas have been subjected to much attention in media, political, policy and scholarly circles. However, many left behind places have existed in their current form, involving entrenched deprivation, for decades (Houlden et al., 2024; Telford and Wistow, 2022). Different governments have implemented various policies to try ameliorate the issues they face; but successful attempts at revitalizing left behind locales have generally been absent (Martin et al., 2021). Such failure is because their decline is an intrinsic part of the neoliberal era (Telford, 2024).

This article explicates how neoliberalism's long-running failure to revitalize the UK's left behind communities is systemically violent in its effects. This is the everyday violence inherent in the capitalist economy and is central to its economic functioning (Ruggiero, 2019); it is regarded as normal and generally accepted by the political class (Evans and Giroux, 2015; Galtung, 1969; ŽiŽek, 2009). Such violence is deeply ingrained and socially injurious, harming the livelihoods of the left behind. As many ultra-realist scholars have also noted, *absence* can be just as important as what is present in our lives (Armstrong, 2025; Hall and Winlow, 2025; Lloyd, 2018; Telford and Lloyd, 2020; Winlow, 2025). The absence of sufficient investment, a progressive alternative political vision and optimism that the future will be better is systemically violent in its consequences. As Winlow and Hall (2019, p. 32) outlined:

“It is not simply presence and action that are causative; so, too, are absence and inaction. The absence of hope, real politics, solidarity and stable and reasonably remunerative employment clearly inform social experience, and the absence of these things are connected to the onward march of neoliberal capitalism.”

This paper is structured as follows. The first section—Left Behind Places—discusses why areas have been left behind in the UK within a political economic context. The next section—Neoliberalism's Failure to Revitalize the Left Behind—explores the decades-long failure to turn around their socio-economic fortunes, zooming in on a range of key policies including the recent Levelling Up agenda. This provides important context for the article's final core section—The Left Behind and Systemic Violence—which briefly discusses some background literature on violence before theoretically explicating systemic violence and absence in relation to left behind places. It closes by suggesting the absence of widespread political engagement and a positive future in left behind zones ensures the continued infliction of systemic violence on the left behind. Whilst there is a burgeoning interdisciplinary literature on these places (for example: Fiorentino et al., 2024; Martin et al., 2021; Pike et al., 2024; Telford and Wistow, 2022; Wenham, 2020), there is a dearth of scholarship that particularly utilizes the theoretical lenses of systemic violence and absence in relation to left behind areas. Accordingly, the paper makes a useful theoretical contribution to debates regarding the conundrum of left behind zones and the enduring difficulties in reviving them.

## Left behind places

A socially intolerable gap between the rich and poor, mass unemployment and destitution especially in industrialized areas in the 1930s, two world wars and support for alternative ideologies, meant the legitimacy of capitalism significantly diminished in the early twentieth century (Hobsbawm, 1995; Martin, 2024). Particularly in light of 6 years of war, death and destruction, the population was exhausted and they demanded radical structural change (Judt, 2010; Marquand and Seldon, 1996). A national focus on social betterment emerged through ensuring full employment, state control of the commanding heights of the economy, a cradle to the grave welfare state and high rates of redistributive taxation served to keep inequalities at reasonable levels (Judt, 2010; Marquand and Seldon, 1996). Such “one nation” politics was combined with a regional policy that sought to shift investment from the economically prosperous and overheated Southeast to lagging Northern areas (Martin, 2024; Pike and Tomaney, 2024), attempting to spread employment opportunities more evenly across the country. Such forms of work often offered a job for life (Marquand and Seldon, 1996), providing longevity and economic stability. As Hobsbawm (1995, p. 257) once noted:

“In the course of the 1950s many people, especially in the increasingly prosperous ‘developed' countries, became aware that times were indeed strikingly improved, especially if their memories reached back to the years before the Second World War. A British Conservative premier fought and won a general election in 1959 on the slogan ‘You've never had it so good', a statement that was undoubtedly correct.”

Living standards persistently improved, with geographical inequalities reaching their lowest level across 1968-early 1970s (Dorling, 2024). In effect, capitalism displayed a remarkable ability to morph into a different variant to regain legitimacy and survive (Hobsbawm, 1995). The above socio-economic restructuring also acted as a *protective scaffold* that transiently contained capitalism's systemic violence, particularly its tendency to create both inequalities and harm (Lloyd, 2018; Raymen, 2022; Ruggiero, 2019). This era, though, was a unique and short phase in its history; it was not representative of its normal functioning (Lloyd, 2018; Streeck, 2016). Structural storms circulated in the 1970s including stagflation, rising public discontent and widespread strike action (Wistow, 2022), culminating in the 1978–79 winter of discontent where over 4 million workers went on strike primarily against below inflation pay rises. As social democracy's validity was questioned, capitalism revolutionized itself once again into a neoliberalised variant.

Neoliberalism's ideals had been forged particularly since the 1938 Walter Lippmann Colloquium in Paris. Prominent scholars gathered to discuss how to ensure governments awarded primacy to market forces, reduced state intervention in markets, curtailed the power of democracy and fractured the working class who they regarded as rather dangerous (Slobodian, 2018). Margaret Thatcher's premiership (1979–90) embodied the UK's first neoliberal phase, unleashing market values such as freedom and entrepreneurialism, trade union reform and decreasing state support for faltering industries (Jenkins, 2007; Wistow, 2022). Whilst neoliberal ideas were once regarded as somewhat outlandish and unrealistic (Marquand and Seldon, 1996; Slobodian, 2018), as we will see, subsequent neoliberal governments extended Thatcher's reforms into areas of society previously untouched by market logic (Telford and Wistow, 2020). Although deindustrialization has a long history and unfolded in a variegated manner across different places, central to neoliberalism has been the transition from a productive to post-industrial economy. Since 1979 most governments have accepted industrial retrenchment alongside the privatization of key societal infrastructure (Christophers, 2023); the advance of asocial individualism (Raymen, 2022); rising inequalities involving an ever-widening economic gap between the rich and poor (Dorling, 2024); a national focus on enterprise and competition rather than collectivism (Telford, 2024); labor market flexibilization (Lloyd, 2013, 2018); and increasingly a new-normal of austerity (Farnsworth and Irving, 2024; MacLeavy, 2024).

Placed in this context, the UK now holds the unenviable status of possessing the worst geographical inequalities out of the advanced economies (Fai and Tomlinson, 2023). Although some people and places have prospered over the past half a century (Telford and Sackey, 2025), many others have been left behind. The term—left behind—is often used by commentators in a catch-all manner, concealing how there are different types of left behind areas spanning urban, rural and coastal as well as neighborhoods, towns, villages and cities (Pike et al., 2024; Telford and Wistow, 2022; Wilson, 2024). However, it speaks to how many of these locales are characterized by social, economic, cultural, political and infrastructural problems (Pike et al., 2024). Such issues include higher than national average rates of crime including violence, unemployment, poorly paid jobs, depopulation especially of its younger and more skilled and educated residents, as well as political dissatisfaction comprising relatively high levels of non-voting (Etherington et al., 2023; Fiorentino et al., 2024; Wenham, 2020; Winlow and Hall, 2022). Such a structural environment can contribute to both relatively high levels of mental illness and deaths of despair (Price et al., 2024; Price, 2025).

Offering a neighborhood categorization of left behind places, Houlden et al. (2024) suggest the most left behind neighborhoods involve either “*entrenched disadvantage*” or “*fluctuating disadvantage*”. These are neighborhoods that have endured long-running deprivation, with deprivation in the latter less severe than in the former, involving more potential to alleviate the issues they face (Houlden et al., 2024). Some of the problems in many of these communities such as high levels of unemployment, criminality, problematic drug use and communal decline are not fleeting predicaments; they have existed for around half a century (Telford, 2024; Winlow and Hall, 2022).

Houlden et al. (2024) also document the geographical spread of left behind neighborhoods, which are principally concentrated in the North and Northeast; in and around core urban areas and deindustrialized and coastal communities. The Local Trust (2020) also revealed how these zones are within places such as County Durham, Middlesbrough (see: Telford, 2024), Hartlepool and Sunderland (see: Winlow, 2001), Hull, as well as Stoke-on-Trent and the old coalfield areas in the Midlands (Etherington et al., 2023). It is important to note, though, that many of these locales often sit close to pockets of affluence (Dorling, 2024). This is because the UK has very high levels of inequality within local authorities (Jones, 2019), with the UK's richest areas also possessing pockets of disadvantage (Telford and Sackey, 2025). Nevertheless, many of the above places possess similar stories of an unjust transition to a post-industrial economy, whereby the economic order that replaced industrialism has not been a sufficient substitute (Martin et al., 2021).

The 2008 financial crisis and the core fiscal response—austerity—also exacerbated the plight of these communities (MacLeavy, 2024) and the systemic violence they endure. As many of these places contain high numbers of residents in receipt of welfare (Beatty and Fothergill, 2020; Gray and Barford, 2018), they were inevitably hit hard by the welfare reforms of the past decade or so (Beatty and Fothergill, 2020; Garthwaite and Bambra, 2018; Gray and Barford, 2018). Partially due to these welfare changes, the UK's poorest households particularly in left behind parts of the Northeast are now more impoverished than the poorest households in countries like Malta and Slovenia (Mosley et al., 2025). Social infrastructure such as high streets, local amenities and transport links also tends to have been badly affected by budget cuts (Tomaney et al., 2024). Crime including forms of violence have also increased in the austerity age (see: Ellis, 2016, 2019).

Although feelings of economic abandonment and political neglect are often widespread in left behind zones (Etherington et al., 2023; Winlow and Hall, 2022), there is also a sense of inertia (Telford, 2022). They are unable to move forward to something new and better. Palpable feelings of nostalgia tend to be present (Winlow, 2025). As we will encounter, some residents often believe that something of great value and importance has been lost (Winlow, 2025). They often take comfort in the memories provided by the certainties and securities of capitalism's post-war age, including the relative togetherness, commonality, camaraderie at work and economic stability that once characterized working class life (Winlow, 2025). Much of this tends to be absent from today's left behind areas, with neoliberalism generating what Winlow and Hall (2013) conceptualized as a *post-social world*. Asocial individualism and competition are now naturalized cultural conditions, resulting in the diminishment of positive values such as obligation, duty and care for one another (Winlow and Hall, 2013). Although the 2008 financial crash, impacts of austerity and rising disaffection have dealt structural blows to neoliberalism, it has proved to be a remarkably resilient form of capitalism (Briggs et al., 2020, 2021).

Whilst this section has discussed left behind places, the article's next section turns to the ongoing failure to resurrect these locales throughout neoliberalism. It then briefly discusses some background literature on violence before explicating how the failure to turn around the socio-economic fortunes of the UK's left behind zones is systemically violent in its socially injurious effects.

## Neoliberalism's failure to revitalize the left behind

As “capitalism unavoidably has socio-spatial inequality structurally and deeply inscribed into its landscapes” (Hudson, 2022, p. 60), there is a long history of governments implementing a range of policies to address geographical inequalities and the problems that beset struggling communities (Jones, 2019, 2024). Martin (2024), for example, highlights how across 1982/83 to 1992/93 state spending on regenerating urban areas increased in real terms by more than 50%. However, this was in response to intense deindustrialization, high levels of unemployment (for instance in 1984, it was 11.9%); a sizable rise in poverty; an associated crime explosion including in violence, heroin consumption and acquisitive forms of criminality (Hall, 2012); as well as growing public unrest including racialized riots in places such as Brixton in London, Toxteth in Liverpool and Chapeltown in Leeds. Although the increased investment did contribute to regenerating some parts of the UK's urban areas (Martin et al., 2021), “regeneration” was principally about encouraging the private sector to innovate and invest, altering the UK's cultural climate away from what was regarded as capitalism's overbearing and inefficient post-war state toward individualism and private enterprise (Parkinson, 1989). Further embodying this was the creation of 38 “Enterprise Zones” across 1981–97. Primarily set up in deindustrializing locales including Swansea, Hartlepool, Corby and Wakefield, these deregulated zones offered a range of incentives to businesses to invest to create jobs (Swinney, 2019). However, they were both costly and somewhat poor at generating new employment, with many other jobs also displaced from different areas (Swinney, 2019).

New Labour's premiership (1997–2010) served to further solidify neoliberalism and the plight of the left behind (Winlow and Hall, 2022). Abandoning its historic social democratic tendencies, the “Blatcherite” (Jenkins, 2007) Labour Party was committed to extending market logic including competitive individualism and privatization into UK society and its institutions (Winlow and Hall, 2022). As Winlow et al. (2015) argued, the Party revised Clause IV which previously committed it to pursuing the collective ownership of the means of production, reformed the welfare state, and awarded primacy to corporate interests. Inequality skyrocketed across their 13-year reign, with the top 1% taking more and more of the national income (Dorling, 2024). In fact, ex- New Labour Cabinet Minister Peter Mandelson claimed they were “intensely relaxed about people getting filthy rich” (Cited in Winlow et al., 2015, p. 69).

Often framed around addressing “social exclusion” in some of the UK's most deprived areas (Durose and Rees, 2012), New Labour also introduced a plethora of initiatives aimed at the neighborhood level. This included the formation of the Social Exclusion Unit in 1997 to try dwindle the disparities in economic prosperity between the most deprived places—the left behind—and the “mainstream”^1^, involving a Neighborhood Renewal Fund that focussed on England's 88 most deprived local authorities (Durose and Rees, 2012). Although these initiatives contributed to a significant reduction in child poverty (McNeil, 2012), too often they focussed on individualized solutions to systemic problems such as the concentration of unemployment, problematic drug use, crime and anti-social behavior in left behind zones, failing to deal with the underlying causes (Durose and Rees, 2012).

A more devolved approach was also adopted through the creation of 9 Regional Development Agencies in England, which were tasked with enhancing regional economic development and regeneration in collaboration with key regional stakeholders (Dalingwater, 2011). However, geographical inequalities generally continued to widen under New Labour's reign, especially between the North and South. As Fuller and Geddes (2008, p. 265) previously remarked:

“New Labour's agenda represents a rejection of the social democratic basis of past social and spatial policies, such as fiscal redistribution or regional policies involving constraints on industrial location, preferring instead to reject any ‘interference' with the market, and downplaying concern with widening social and economic inequalities”.

Throughout neoliberalism, governments have generally focussed on cozying up to market forces, often suggesting they can do little to address ever-widening inequalities (Hudson, 2022). Accordingly, areas that are ‘left behind' tend to stay behind' (Houlden et al., 2024, p. 7). This was clear across 2010–19 with various Conservative administrations who implemented austerity in light of the 2008 financial crisis. Such measures, as mentioned, were not geographically neutral and had a debilitating impact on left behind places and many of their residents (Gray and Barford, 2018; MacLeavy, 2024). As Atkins and Hoddinott (2020) pointed out, across 2009/10 to 2019/20 local councils' key income stream in the form of government grants were cut from £46.5bn to £28bn. Such a reduction in expenditure was felt acutely in left behind zones, since low-income groups tend to rely more upon public services including welfare support.

The Conservatives and Liberal Democrats Coalition government (2010–15) also replaced Regional Development Agencies with 39 Local Enterprise Partnerships (LEPS) in England. Embodying a move away from mainly focussing on urban regeneration, LEPS involved an increased focus on local economic growth led by the private sector in collaboration with the public sector (Jones, 2019). However, they failed, partially as they were rolled out during a period of unprecedented budget cuts, spiraling inequality and were not statutory bodies (Pike et al., 2015). As Jones (2019) noted, this meant they lacked both the resourcing and power to address localized problems.

Further policy changes ensued, though, with a Northern Powerhouse agenda announced in 2014. This involved a vision to better connect the North's key cities and devolve power to mayoral combined authorities, partially to improve both economic opportunities and growth across the North (Centre for Cities, 2015). The first six metro mayors were elected in 2017, including Andy Burnham in Greater Manchester and Ben Houchen in the Tees Valley. However, inequalities between the regions continued to magnify, partially in light of both austerity and how the Northern Powerhouse agenda was underpowered (Raikes and Johns, 2019). Across 2009/10 to 2017/18 public spending for the North declined by £3.6bn, yet for the Southeast and Southwest combined it increased by £4.7bn (Raikes and Johns, 2019). Writing on the seeming futility of various policies to significantly address the plight of left behind places, Martin et al. (2021, p. 96) indicated that:

“longstanding problems have persisted and cases of places able to turnaround their prospects are rare. At best, such policies may have only prevented geographical inequalities from worsening at a faster rate.”

Perhaps the most notable policy to try and resurrect left behind areas under neoliberalism was the Conservative government's (2019–24) “Levelling Up” agenda. Announced in 2019 alongside the electoral slogan of “get Brexit done” by Boris Johnson, the idea attracted widespread support across the “Red Wall”—historically Labour voting constituencies ranging from South Wales to Blyth Valley in Northeast England that turned blue in 2019 often for the first time in their history (Winlow and Hall, 2022). Levelling Up was regarded as catchy but rather ambiguous terminology that spoke to the need to address the misfortunes of the UK's most deprived communities (Telford and Wistow, 2022). Details on how to achieve this were rather sparse—however, that somewhat changed with the publication of the Levelling Up White Paper in 2022.

Cast as probably the most important spatial policy document in nearly a century (Martin et al., 2022), the White Paper revealed 12 medium-term missions to accomplish by 2030 (HM Government, 2022). As such, the document acknowledged that it would take time to address inequalities, and that success cannot be achieved overnight. These missions included increasing wages, employment and productivity across the UK; enhancing local public transport so it is closer to London's standards; ensuring 5G broadband is available to most of the UK citizenry; narrowing the gap in wellbeing between the most affluent and left behind places; bolstering public investment in research and development by around 40% outside the South East; considerably increasing the amount of primary school children achieving the expected level in reading, writing and mathematics; and ensuring more citizens complete high-level skills training especially in lower skilled localities (HM Government, 2022).

Progress on Levelling Up, though, was described as ‘glacial', with the nation as a whole regressing in relation to the missions (Farquharson et al., 2024). Key criticisms that shaped this failure include Levelling Up did not challenge the dominance of London and its contribution to geographical inequalities, including through receiving a disproportionate share of public investment (Martin and Sunley, 2023); the allocation of funds involved a top-down process of local authorities competing against one another for small pots of infrastructure funding that wasted both time and resources (Atherton and Le Chevallier, 2023); it failed to offer transformative policies that would help to undermine neoliberalism (Telford and Wistow, 2022); it was not a well conceptualized policy programme but political opportunism to appease first time Conservative voters in the Red Wall (Hudson, 2022); and it was significantly underpowered in relation to the scale of the problem (Martin et al., 2021 2022). As the Labour Party regained political power in 2024, they sounded the agenda's death knell by abandoning it within a week of winning office, with the Deputy Prime Minister Angela Rayner describing it as a “gimmick” (Telford, 2024).

Levelling Up formed yet another example of a failed, short-term policy (Coyle and Muhtar, 2023). As indicated, most governments have been dedicated to neoliberalism's political economic restructuring, while many of the above policies have been consistently underpowered in relation to the scale of inequality. This is because the UK has been a low investment nation throughout much of the neoliberal era (Telford, 2024). As Dibb and Jung (2024) highlight, the UK has possessed the lowest rate of investment in the G7 for 24 of the past 30 years. Public investment is also short-termist in relation to comparable nations, which stifles private investment (Dibb and Jung, 2024). This is important since investment is the fuel that powers an economy and helps to address deficiencies in infrastructure, equipment and productivity that also contributes to increases in wages.

Such low investment is linked to “fiscal rules”. Whilst these were first introduced by New Labour in 1997, since 2010 they have frequently changed and increased in governmental importance (Telford, 2024). These rules tend to be short-termist (Telford, 2024), not least as the current ones focus on reducing debt as a percentage of GDP and do not factor in investment with returns beyond the electoral cycle of 5 years. Given the above, it might be more accurate to regard UK neoliberalism's long-running inability to revive left behind areas as the “governance of failure” (Jones, 2024, p. 56), which is an inherent part of neoliberal capitalism. After briefly discussing some background literature on violence, the next section explicates how the policy failure documented above is systemically violent in its effects.

## The left behind and systemic violence

The headlong stream is termed violent,

but the riverbed hemming it in is

termed violent by no one.

The storm that bends the birch trees

is held to be violent

But how about the storm

that bends the back of the roadworkers?

(On Violence by Bertold Brecht, playwright and poet,

1898–1956).

When thinking about violence, we tend to focus on its most visible forms. This includes the recent increase in retail-based violence and outbursts of alcohol fuelled violence within the night-time economy (Bushell, 2023; Winlow and Hall, 2006; Bushell and Braithwaite, 2024). The latter form of violence is normalized and largely perpetrated by men, often characterized by spontaneity, excitement and barbarity (Ellis, 2016; Winlow, 2001). It also includes violence against women, with a large body of feminist research significantly contributing to our understanding of one of the globe's most persistent criminological problems (see: DeKeseredy, 2025). Drawing on ethnographic work with violent men in a deprived locality, Ellis (2016) suggests there is a culturally mediated link between trauma in childhood and a commitment to violence later in life. This could involve a cold-hearted father who is physically violent toward his son, reminding him that the world is a difficult place, and he must be tough and ready to tackle the threats that will inevitably emerge in the future. Feelings of shame, humiliation, and helplessness can often congeal, manifesting in the son engaging in violence in later life often to avoid these powerful and negative emotions. For Ellis (2016), then, violence is often the product of the interlinked forces of biographical experience, emotion, memory, culture and the political economy. Other forms of visible violence include homicide and the *crime of all crimes*—genocide—which is often driven by historical factors, religion, ethnicity and nationality (Soundararajan et al., 2024).

It is incumbent upon scholars, though, to move away from the foreground and focus upon the background (Hall and Winlow, 2025). In his seminal work on peace, conflict and violence, Galtung (1969) noted that focussing solely on visible violence is a rather narrow conceptualization and he encouraged scholars to broaden their gaze. This particularly included toward “structural” or “indirect” violence that is ingrained within the social fabric. Such violence can seem “about as natural as the air around us” (Galtung, 1969, p. 173), yet it often inflicts far more damage than interpersonal violence. Bufacchi (2005, p. 198) also asserted that viewing violence solely as committed by an individual involving force and the intention to harm is a “*minimalist conception*”, whereas broadening the analytical lens toward structural violence forms part of a “*Comprehensive Conception of Violence*”. For Bufacchi (2005) and Ruggiero (2019), this can involve the violation of human rights including the failure to satisfy basic human needs, often resulting in insecurity and instability feeling like naturalized conditions of existence, rather than tethered to neoliberalism (Evans and Giroux, 2015).

Offering an updated tripartite framework, ŽiŽek (2009) focusses particularly on subjective, symbolic and systemic violence. Subjective violence refers to acts involving clearly identifiable perpetrator(s) and victim(s), whereas symbolic violence embodies the violence inherent within language and its various modes. For ŽiŽek (2009), the causes of subjective violence are often wedded to capitalism's systemic violence. This is the violence internal to the capitalist system. Such background violence is regarded as natural and inevitable; it is central to the current state of things (Ruggiero, 2019; ŽiŽek, 2009). Writing on capitalism and the gradual nature of environmental breakdown, Nixon (2011, p. 2) outlines the related concept of “slow violence”:

“By slow violence I mean a violence that occurs gradually and out of sight, a violence of delayed destruction that is dispersed across time and space, an attritional violence that is typically not viewed as violence at all. Violence is customarily conceived as an event or action that is immediate in time, explosive and spectacular in space, and as erupting into instant sensational visibility. We need, I believe, to engage a different kind of violence, a violence that is neither spectacular nor instantaneous, but rather incremental and accretive, its calamitous repercussions playing out across a range of temporal scales.”

As mentioned, the predicaments of the UK's left behind communities have unfolded in a gradual manner over the past half a century or so. Whilst inequalities between places were kept at reasonable levels during capitalism's post-war social democratic phase (Hall and Winlow, 2025), “neoliberalism's slow-motion social dislocation” (Telford and Wistow, 2020, p. 553) has involved a sequence of processes that eviscerated once relatively prosperous and functional areas. This particularly includes the *toxic tripartite* of industrial collapse, austerity and relatively low levels of investment, resulting in these places losing their raison d'être and enduring social problems especially relatively high levels of unemployment, poverty and crime.

These issues have violent consequences (Telford and Lloyd, 2020). Many residents of left behind zones often suggest they are undesirable places to live as they blight the livelihoods of people living there (Telford, 2022; Winlow, 2025). As the Social Mobility Commission (2024, p. 109) reported, the “least favorable conditions of childhood” tend be found in left behind areas in the North such as Middlesbrough, Oldham and Stoke-on-Trent. Parts of these places diminish an individual's quality of life, reducing their opportunities to access good education, attain well-paid work and achieve upward social mobility (Social Mobility Commission, 2024). Systemic violence contributes to residents' subjective feelings of left-behindness, with many people claiming they have been economically abandoned (Winlow and Hall, 2022).

Such systemic violence is wedded to how left behind areas are deemed politically invisible; too often policies have formed mere sticking plasters to entrenched issues and have been hugely under resourced. For example, as Telford (2024) notes, the Levelling Up funding of around £46.9mn that left behind Middlesbrough received was only £9.4mn more than the £37.5mn the local council lost through austerity across 2013–24. This funding was hardly enough to restore the level of resourcing that was in place before 2013 when Middlesbrough was already one of the UK's most left behind areas and had long endured structural violence (Lloyd, 2013; Price et al., 2024). Despite the initial burst of optimism and hope that Levelling Up generated in some left behind places (Telford, 2023), it is evident that neoliberalism's political class do not regard them and their residents as worthy of the resourcing required to address the problems they face. Violence flows through this underinvestment, since it ensures the prolongation of the left behind.

Systemic violence is also intrinsic to what Evans and Giroux (2015, p. 15) term the “*politics of disposability*”. Individuals, families and the neighborhoods that occupy left behind zones are regarded as excess to be disposed of; they are not economically required and are often cast as a burden on a nation's resources. Ensnared in precarious and cyclical forms of work, economically insecure residents endure everyday forms of hardship that erodes their social status and stake in society (Evans and Giroux, 2015). This means both left behind areas and their inhabitants lose their sense of purpose and can be politically disposed of. Evans and Giroux (2015) suggest they then become targets for state surveillance, while policies are implemented including the erosion of welfare which heightens their difficulties.

Evans and Giroux (2015) assert that this violence makes life unbearable for some residents, with evidence supporting this claim. Across 2017–19 in the most deprived 10% parts of England, the suicide rate was 14.1 per 100,000 people which is nearly double the level of 7.4 per 100,000 people in the least deprived 10% of localities (Kirk-Wade, 2025). Accordingly, systemic violence engenders enclaves of suffering in left behind locales, with particularly men lacking an identity and fulfillment (see: Gibbs et al., 2022; Price, 2025). Further elucidating this is how, as of February 2025, over double the amount of people (291,083) in the highest deprived decile were in contact with mental health services in comparison to the least deprived decile (137,725) (NHS Digital, 2025). Data also revealed that, across 2015/19, the top three places with the highest rates of antidepressant prescriptions were left behind Blackpool, Sunderland and Barnsley (Lalji et al., 2021). Such a higher prevalence of psychic despair in impoverished zones is generally consistent over time—for instance across 2022/23, 39.8% more people in England's most deprived places were prescribed antidepressants compared to the least deprived areas (NHS Business Services Authority, 2023).

Focussing on the lives of three young people and their paths to violent radicalization, Bhutto's (2019) novel *The Runaways* discusses how violence is systemically toxic and infiltrates peoples' lives, sentiments and outlooks. She suggests violence is *atmospheric* and can often be both seen and felt. As evidenced above, the systemic violence of neoliberalism creates psychic misery. Many residents of left behind areas have lost hope that their circumstances will improve, falling into a state of despondency. Neoliberalism's politics of disposability is based on the premise that individuals occupying left behind areas have simply not worked hard enough and have nobody else to blame but themselves for their problems (Evans and Giroux, 2015). Such individualization has a long history but is a key feature of neoliberalism (Raymen, 2022; Winlow and Hall, 2013), and it seeps into approaches to addressing mental ill health. Rather than focussing on tackling systemically violent conditions, the onus is on the individual to adapt, change and overcome the issues they face (Fisher, 2009, 2018). For example, several government ministers have recently remarked that schoolchildren need more “grit” to help alleviate their mental ill health (see: Wood, 2025). However, without addressing the systemic violence of neoliberal capitalism, the prevalence of mental ill health especially in left behind zones will inevitably persist.

Systemic violence is also evident in how cancer-related deaths are nearly 60% higher in the UK's most deprived localities compared to the least deprived (Cancer Research UK, 2025). This is due to a wide range of issues, including how left behind areas possess higher rates of risk factors such as smoking, obesity, poor diets as well as less exercise (Munford et al., 2022). However, it is also related to how people from the most deprived neighborhoods wait longer before seeking help, partially as they worry healthcare professionals will not take their symptoms seriously (Cancer Research UK, 2025). They are also more likely to endure longer waiting times for starting treatment and are even less likely to receive treatments for certain forms of cancer (Cancer Research UK, 2025). As such, it is evident that neoliberalism's systemic violence infiltrates the core of the left behind and detrimentally impacts upon their neuropsychology.

A higher prevalence of psychic distress and cancer-related deaths are tethered to how systemic violence is also felt in the lower levels of healthy life expectancy in left behind places. The Health Foundation (2025a) recently outlined how the healthy life expectancy of women in England's most deprived communities is 51.9 years, in contrast to 70.7 years in the least deprived zones. The difference for men is 18.2 years. Accordingly, the average gap in healthy life expectancy between the UK's most and least deprived locales is 19 years (The Health Foundation, 2025a). This inequality is starker at a more local level. As The Health Foundation (2025b) also documented, the relatively affluent local authorities of Rutland (74.7 years), the Orkney Islands in Scotland (71.2 years) and Wokingham (70.9 years) contain the highest male healthy life expectancy at birth. These zones, though, are in sharp contrast to left behind Inverclyde (54.4 years), Blackpool (53.5 years) and Kingston upon Hull (53.8 years), who contain the lowest levels of male healthy life expectancy. Such inequalities are rather persistent and worsened considerably across 2010–20 (Marmot, 2020), revealing how the systemic violence of neoliberalism was amplified in the austerity era.

As ultra-realist scholars have mentioned, it is not simply what is present that is significant, but also what is absent (Armstrong, 2025; Hall and Winlow, 2025; Telford and Lloyd, 2020; Winlow and Hall, 2019). Absence can have a considerable bearing upon our lives; it can be viscerally felt in a left behind locality (Telford, 2022). People can feel as though something important, which may have once offered stability, security and inspire hope of a better future, is no longer present (Winlow, 2025). As Frers (2013) remarked, becoming absent can happen suddenly or unfold over time. It can engender a panoply of strong and negative emotions (Frers, 2013), including insecurity and fear. Experiencing absence is also shaped by the connection to what has been lost, with the loss of a particular place one of the most palpable forms of absence an individual can endure (Frers, 2013). As such, the nagging sense that the presence of something that was once of great value has been lost can be deeply troubling.

Characterizing UK politics throughout much of the neoliberal era has been the absence of authentic political representation in left behind places (Winlow et al., 2015; Winlow and Hall, 2022). Many residents have long believed that no matter who is in power, the fundaments of existence remain the same (Telford, 2022). Evans (2020) and Ruggiero (2019) highlight how one of the myths of violence that helps to sustain it is that it is natural and unavoidable. The long-running decline of left behind places under neoliberalism has often been regarded as a fait accompli, with politicians occasionally claiming high levels of inequality is inevitable within a marketized economy (Hudson, 2022). This shapes an absence of belief that left behind zones can be revived, not least as the resources required to resurrect them are not forthcoming under neoliberalism (Telford, 2024). In consequence, politics becomes a source of cynical dissatisfaction. Although the left behind desire positive structural change in their areas, the absence of political representation ensures that this is forestalled.

Such absences often result in the left behind cynically withdrawing from politics. Many left behind residents see no value in engaging with the political system, suggesting politicians serve their own interests and do not care for people like them (Telford and Wistow, 2020). Whilst declining voter turnout at elections has been a key feature of neoliberalism (Winlow et al., 2015), it has been particularly acute in left behind places. At the 2024 general election, for example, the Labour Party were elected on what has been widely regarded as a *loveless landslide*. Only 52.8% of the voting age population voted; the lowest share of the citizenry to vote since it became permissible for both men and women in 1928 (Patel and Valgarosson, 2024). This means that if non-voters were a political party, they would have been the biggest party by far (Patel and Valgarosson, 2024). Such absent interest was clear in many left behind zones as voter turnout was often very low—for instance, Hull East (42.2%), Blaenau Gwent and Rhymney in Wales (42.7%), Blackpool South (45.4%), Stoke-on-Trent Central (47.8%), Rotherham (48.6%), Easington (49.5%), Hartlepool (49.7%), Middlesbrough (54.1%) and North Durham (56.8%).

Whilst these places could once look to the future with hope and optimism, they are now also characterized by the absence of a positive future. Drawing on ethnographic research with working class people in a post-industrial Northern city, for instance, Winlow (2025) notes there was a fatalistic sense that the future was inevitably going to be worse than the past and present. The negative developments of the neoliberal epoch, including the diminishment of remunerative employment and increasing disorderliness of post-social life, were going to continue. The idea of gradual improvement in these types of places has long collapsed (Winlow, 2025). Only further decline and sadness await. Many people felt as though they had no control over this descent; they were passive spectators of crumbling locales that had no place in the future. Writing on this absent future, Winlow (2025, p. 42) asserted that:

“The future increasingly appears to us as an imponderable abyss of which we can know nothing. No clear signs of progress and improvement can be discerned. We appear to have lost the capacity to project images of bountiful new freedoms onto the darkness of this abyss. We no longer assume that future technologies will heal the natural environment and solve the world's ills. The field of politics no longer equips the people with coherent images of a positive future we can work towards and nor does it offer us feasible alternatives to what already exists.”

This can result in the loss of hope and positivity about one's position in the world. It can contribute to people attempting to move away from the area their family has called home for centuries in search of employment, resulting in the historical severing of tradition and the ties that have rooted people to particular places (Winlow, 2025). The atmospheric nature of this violence (Bhutto, 2019) can engender a palpable sense of dejection, with many left behind residents believing that poorly paid work, decaying infrastructure and communal degeneration are just how things are today. In effect, as Fisher (2018) remarked, *the future has disappeared*.

The absence of political representation, engagement and a positive vision of the future are connected to the continuation of neoliberalism's systemic violence. These absences can be socially injurious, making it more expedient for neoliberalism's political class to focus on facilitating the interests of market forces rather than resurrecting left behind places. Politically paralyzed by cynicism and fatalism (Winlow, 2025), the inhabitants of left behind areas thus inadvertently contribute to the plight of their localities through withdrawing from politics and aiding the election of neoliberal governments. Such disengagement also serves to crystalize the negative ideology of capitalist realism. This is the pervasive mood in UK society, especially in left behind areas, that there is no alternative to the present political economy and its hollowed-out form of politics (Fisher, 2009, 2018). As any other political economy is deemed unrealistic (Fisher, 2018), all that left behind residents can do is individualistically accept and adapt to the inequities of neoliberal capitalism. As such, the absence of pervasive political participation and a positive future within the “dying social worlds” (Hall and Winlow, 2025, p. 245) of the left behind contributes to the reproduction of their misfortunes and the systemic violence they endure.

## Concluding thoughts

In the immediate decades after World War Two, the UK became more socio-economically equal in comparison to the decades that preceded the war (Hobsbawm, 1995). A range of policies including a relatively generous welfare state, governmental control of key public utilities, a national health service free at the point of use and a regional policy focus on diverting investment to less prosperous locales raised the living standards of the working class in an unprecedented way (Telford and Lloyd, 2020). Shifting to neoliberalism in the late 1970s, though, gradually unraveled the social improvements of the post-war age. Political primacy to market forces, deindustrialization, the privatization of core utilities, the ascent of atomized individualism and ongoing austerity, in particular, have contributed to the UK falling apart at the seams (Dorling, 2024; Wistow, 2022). Defined by entrenched structural problems including deprivation, crime, insecure jobs, political disaffection, outmigration and low levels of educational attainment and skills, left behind places are central to what is now the most geographically unequal nation out of the advanced economies (Fai and Tomlinson, 2023).

Throughout neoliberalism, governments have implemented a range of policies to try and address geographical inequalities and the plight of the left behind (Jones, 2019, 2024; Martin, 2024). Although there are instances of these policies having a positive impact, often they have formed mere sticking plasters to ingrained issues. The most recent Levelling Up agenda, for instance, was particularly hampered by significant underinvestment especially in light of the damage austerity wrought on left behind communities (MacLeavy, 2024; Telford and Wistow, 2022). The long-running empirical evidence is also rather damning—examples of left behind zones turning around their socio-economic predicaments are scarce (Martin et al., 2021). This is principally because their decline is internal to neoliberalism's political and economic arrangements (Telford, 2024).

Whilst we tend to focus on the most visible forms of violence, if we widen our conceptual focus away from the foreground to encompass the background, we encounter a potent and more systemic form of violence that has rather pernicious consequences. A violence that is central to our present political and economic system (Ruggiero, 2019; ŽiŽek, 2009). One of the worst victims of UK neoliberalism's systemic violence, left behind places have endured persistent structural injuries for nearly half a century. Long regarded as politically disposable (Evans and Giroux, 2015), many left behind residents have lost a sense of purpose and identity (Price, 2025). This has contributed to a higher concentration of psychic gloom in these areas, with life being intolerable for some individuals (Price et al., 2024). It is not simply what is present in left behind zones that is important, though, but also what is absent (Winlow, 2025). The absence of widespread political representation, engagement and a positive future among the left behind ensures the prolongation of neoliberalism's systemic violence.

Writing on the slow violence of global warming, Nixon (2011) noted that violence can be exponential as its impact is felt both in the short and long-term. It has a domino effect, with slow-motion environmental breakdown resulting in soil degradation, food insecurity, resource wars, erratic weather patterns and the loss of biodiversity. The systemic violence of neoliberalism is also potentially generative, solidifying the conditions that make subjective violence more likely (ŽiŽek, 2009). This includes trauma, distress, anger, unemployment and the absence of hope (see: Ellis et al., 2021). Providing a clear case in point is the increase in subjective violence during the era of austerity (Ellis, 2019).

The feelings above can thus often manifest in inarticulate rage (ŽiŽek, 2009), which was also evident in the UK in the summer of 2024. In the wake of the Southport stabbings, including the murders of three young girls aged 6, 7, and 9 by Axel Rudakubana, riots plagued various left behind zones including in parts of Middlesbrough, Rotherham, Sunderland, Blackpool, Hartlepool, Hull and Stoke-on-Trent. Whilst racism, xenophobia and social media all inevitably played a part, neoliberal politicians including the Prime Minister, Keir Starmer, were keen to frame the riots as mere “far-right thuggery” (see: BBC, 2024). However, it is difficult to ignore the linkages between areas with high deprivation and engagement in rioting (Duncan et al., 2024). As mentioned, many of these places are long-running victims of systemic violence, with their residents politically and economically abandoned throughout neoliberalism.

Despite neoliberalism's protracted infliction of systemic violence on the left behind, this form of political economy still retains a degree of what Hall and Winlow (2025) recently termed “emotional credibility”. This is an embodied belief misrecognised as objective knowledge, with a sizable rump of the citizenry still feeling that neoliberalism is somewhat credible. Such credibility is tied to the potency of capitalist realism. As all alternative forms of organizing the economy and society have been ideologically defeated there is currently nowhere else for the left behind to go; no other political and economic system to identify with (Hall and Winlow, 2025). Through continuing to elect neoliberal governments (Telford, 2022), the public is informed that things will gradually get better as all we need to do is continue to put a modicum of faith in the system. This feels far more *credible* to the left behind than joining with other disadvantaged people in a collective struggle, which they often regard as both pointless and unrealistic, to pursue structural change (Hall and Winlow, 2025). A considerable amount of the citizenry, especially those in more affluent places, also find it emotionally credible—believable—that residents of left behind zones are at least partially responsible for their plight. Overcoming this emotional disdain for both the left behind and a more collectivized political economic project is essential if we are to significantly reduce the systemic violence they endure. However, in the immediate future at least, it feels far more *emotionally credible* that neoliberalism's violent consequences will continue to ripple through the UK's left behind places.

## Footnotes

^1^See Winlow and Hall (2013) for a critique of the idea of social exclusion.
